# Experience of an endocrine surgeon in laparoscopic transperitoneal adrenalectomy

**DOI:** 10.1186/s12893-019-0599-0

**Published:** 2019-09-11

**Authors:** Serkan Teksöz, Bekir Burak Kılboz, Yusuf Bükey

**Affiliations:** 1 Istanbul Universitesi Cerrahpasa Tip Fakultesi, General Surgery, Istanbul, Turkey; 2 Istanbul Universitesi Cerrahpasa Tip Fakultesi, Istanbul, Turkey

**Keywords:** Laparoscopy, Training, Learning curve, Endocrine

## Abstract

**Background:**

Laparoscopic adrenalectomy (LA) is currently recognized as the gold standard for the treatment of most adrenal lesions, with a high safety and feasibility profile. This study aimed to present the extensive experience of a specialized endocrine surgeon in LA in a relatively large series of patients.

**Methods:**

A total of 116 LAs performed from June 2009 to 2018 were evaluated in terms of adrenal pathologies, perioperative management, complications, conversions, tumor size, operative time, and learning curve. The learning curve was assessed using the cumulative sum (CUSUM_OT_) technique.

**Results:**

Of 116 LAs, 107 (92.2%) were completed successfully, 77 (72%) of which were for Cushing’s syndrome (*n* = 43, 55.8%), pheochromocytoma (*n* = 26, 33.8%), and Conn’s syndrome (*n* = 8, 10.4%). Conversion was required in 9 cases (7.8%), the most common cause being limited space complicating dissection (*n* = 3). The mean operative time for successful LAs (unilateral 85, bilateral 22) was 74.7 min (range 40–210 min) and the mean hospital stay was 1.7 days (range 1–5 days). Gender, tumor size and body mass index were found to have no significant relationship with the operative time (*p* > 0.05). Postoperative normalization in hormone profiles was obtained in all patients but one. Aside from grade-I port-site infections in four patients (3.7%), no postoperative major complications and 30-day mortality were observed. On the CUSUM_OT_ graph, the learning period covered the first 34 operations.

**Conclusions:**

Laparoscopic adrenalectomy is safe and advantageous, but requires a dedicated team involving experienced endocrine surgeons who have achieved competency after completion of the learning curve.

## Background

Laparoscopy undoubtedly represents a “revolution” in modern surgery, providing great advantages especially for the part of the patient. It has also proved to be very effective in some major surgeries (adrenalectomy, nephrectomy, colectomy, splenectomy, etc.) and to be the treatment of choice in most cases [1].

The transition to laparoscopy first began in dedicated surgical centers, without any standardized training programs on how to adopt this technology and with difficulties for surgeons to learn how to safely use this new technology during specialization and fellowship training [2].

For laparoscopic adrenal surgery, several surgical approaches have been described including the transperitoneal, lateral retroperitoneal and posterior retroperitoneal approaches [3]. Each of these techniques is highly successful in experienced hands and surgeons are strongly recommended to choose the approach they are most skilled in and familiar with. Currently, the laparoscopic transperitoneal approach is accepted as the standard procedure due in part to its common use [3]. However, adrenalectomy is not a common procedure and requires a learning curve of about 30 cases [4, 5]. Interestingly, more than 90% of all adrenalectomies are performed by surgeons whose endocrine operations account for less than 25% of their surgical practice. On the other hand, surgeons whose endocrine operations account for more than 75% of their surgical practice perform only 3% of adrenalectomies [6]. More strikingly, 50% of all adrenalectomies are performed by surgeons whose annual adrenalectomy operations total only one or two [6]. These figures not only reflect the urgent need for intensive training of residents engaged in endocrine surgery but also the sine qua non role of dedicated centers to perform adrenalectomy operations.

The growing concern about training of those who want to specialize in endocrine surgery and about the acquisition of sufficient experience in the field of laparoscopic adrenal surgery was the major trigger for the present documentation of extensive experience of a single endocrine surgeon with laparoscopic transperitoneal adrenalectomy.

All reasons for doubt have been eliminated about the feasibility of laparoscopy in adrenal tumors associated with hypersecretion such as pheochromocytoma, as well as in large masses and secondary malignancies [7]. Some debate still exists on the use of laparoscopy in primary adrenal carcinoma, although many surgeons prefer laparoscopy as long as feasibility criteria are met [8–10]. Leaving aside adrenocortical carcinoma, we propose laparoscopic adrenalectomy for metastatic adrenal cancers and large adrenal masses with no evidence of malignancy provided that it is duly performed.

The present study aimed to assess a relatively large series of patients who underwent laparoscopic and open adrenalectomies, with particular focus on complications, the reasons for conversion to open surgery, and issues to be considered in patient selection.

## Methods

**Fig. 1:**
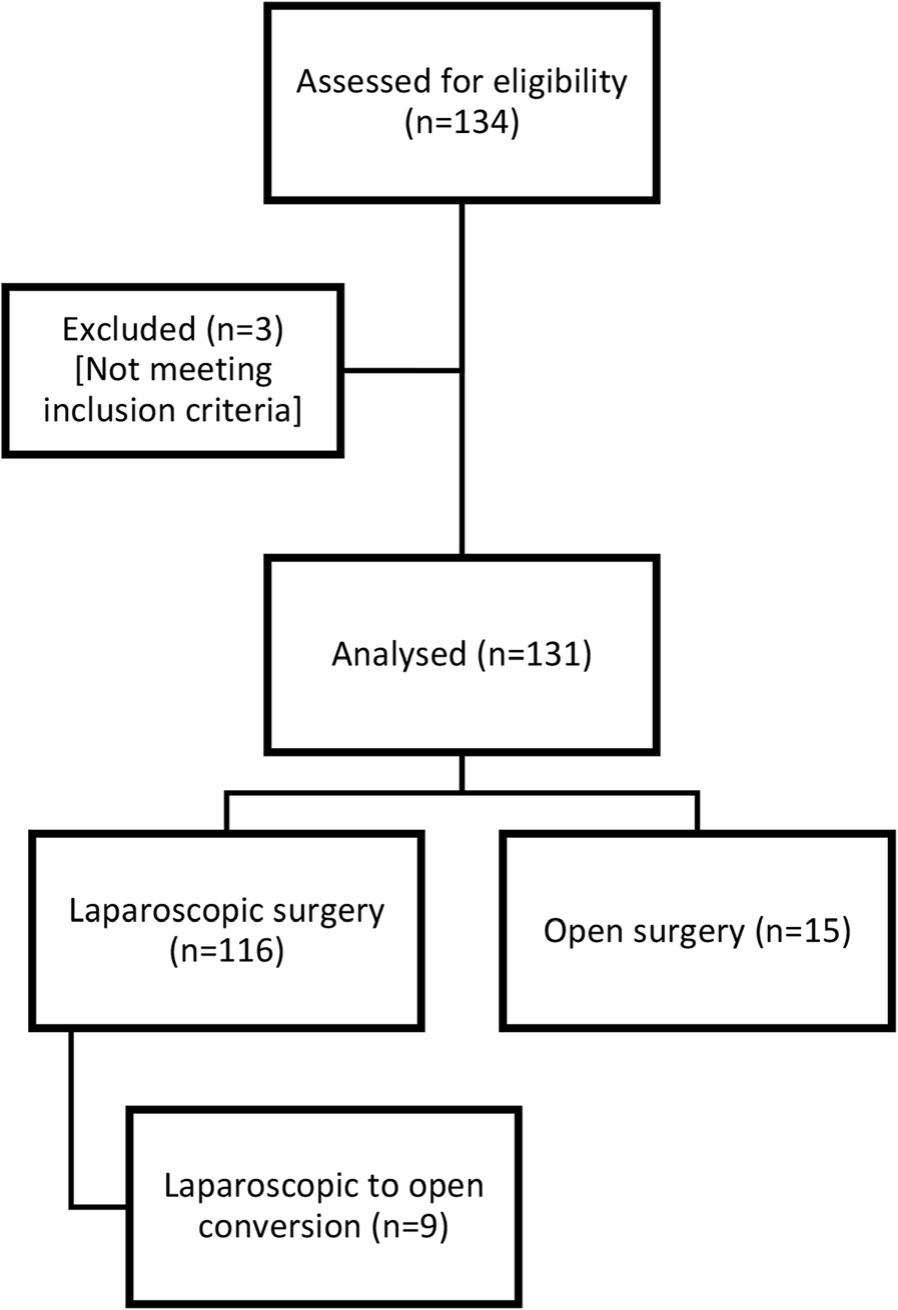
Study population

This retrospective study included all patients who underwent laparoscopic or open adrenalectomies at a single university hospital from June 2009 to 2018. All data about the patients and operations were prospectively inputted into the database of the hospital. All surgical interventions were performed by the same endocrine surgeon (ST). Most of the patients were referred by local endocrinology units. The operations were performed in the Endocrine Surgery Unit of the Department of General Surgery, Cerrahpaşa Medical Faculty, İstanbul University. The study was approved by the Clinical Research Ethics Committee of Cerrahpaşa Medical Faculty (Registration no: 83045809–604.01.02). Consent has has been obtained from each patient or subject after full explanation of the purpose and nature of all procedures used. Patients who had simultaneous surgeries such as cholecystectomy (*n* = 2) or incisional hernia (*n* = 1) were excluded from patient population (Fig. 1).

### Preoperative procedures

To obtain a blood pressure of 120/80 mmHg in patients with pheochromocytoma, α-adrenergic blockade was initiated with phenoxybenzamine at an initial dose of 10 mg (mean 100 mg, maximum 200 mg) starting 14–21 days before the operation. To prevent orthostatic hypotension associated with the α-adrenergic blockade, a high sodium diet along with intravenous fluids was started after the second or third day of medical treatment. In the presence of tachyarrhythmia, a ß-adrenoreceptor blocker was administered in addition to the α-adrenergic blocker therapy.

### Laparoscopic surgical technique

All patients underwent surgery under general anesthesia, with orotracheal intubation and antibiotic prophylaxis. A lateral transabdominal approach was used for all laparoscopic adrenalectomies. Patients were placed in the lateral decubitus position, with the tumor-side up and a cushion placed underneath, giving an angle of 50–60 degrees to the operation table. Two 10-mm and one 5-mm trocars (Medtronic) were used for left and right adrenalectomies. An additional port on the right was necessary to place a liver retractor. A 30-degree 10-mm optical laparoscope was used and dissection was performed using a Ligasure™ (Medtronic) energy-based device. Additional ports were used in obese patients or when necessary. The excised specimen was placed in a retrieval bag (EndoBag™, Medtronic) and removed with minimal expansion of the initial incision site.

### Data for evaluation

Patient- and surgery-related data included demographic features, operation indications, comorbidities, body mass index (BMI), intraoperative parameters, tumor size, conversion to open adrenalectomy, and morbidity as defined by complications that were classified using the Clavien-Dindo system [11].

### Statistical analysis

Retrospective data were entered into a spreadsheet (Microsoft Excel, 2017) and then processed. Normal distribution was not observed in the Kolmogorov-Smirnov test. In addition to standard statistical calculations, the Spearman’s correlation, Kruskal-Wallis H and Mann-Whitney U tests were performed to determine intergroup differences and evaluate relationships between patient- and operation-related variables. Moreover, in accord to central limit theorem, Independent t-test and Pearson’s Correlation Co-efficient were also used. Statistical analysis was performed using the SPSS version 25.0 (IBM SPSS Statistics for Macintosh, 2017, IBM Corp. Armonk, NY). A *P* value of less than 0.05 was considered significant.

### Cumulative sum analysis

The cumulative sum (CUSUM) technique is a time-trend graphical method of determining trends in changes over time for sequential data, particularly on competence acquisition, on a case-by-case basis by calculating the sequential difference between raw data and means. For this analysis, cases were chronologically ordered from the earliest to the latest. The CUSUM of the operative time for each case (CUSUM_OT_) was calculated using the formula CUSUM_OT_ = ∑^n^_i = 1_ (x_i_ − μ), with x_i_ denoting the operative time for a particular case and μ the mean duration of the entire sequence. Then, a chronologically plotted CUSUM chart was obtained. This method was previously used to evaluate the learning curve for surgical operations [12].

## Results

A total of 131 patients (91 females, 69.5%) underwent laparoscopic (*n* = 116, 88.5%) or open (*n* = 15, 11.5%) adrenalectomies from June 2009 to 2018 for the following conditions: Cushing’s syndrome (*n* = 52, 39.7%), pheochromocytoma (*n* = 32, 24.4%), incidentaloma (*n* = 29, 22.1%), Conn’s syndrome (*n* = 8, 6.1%), cystic lesions (n = 5, 3.8%), metastatic disease (n = 2, 1.5%), and other adrenal pathologies (n = 3, 2.3%). The mean age of the patients was 48.1 years (range 23–72 years), and the mean BMI was 29.1 kg/m^2^ (range 18.8–43.0 kg/m^2^). Of 116 laparoscopic adrenalectomies, 107 (92.2%) were completed successfully without the need for conversion, 77 (72%) of which were for functional tumors including Cushing’s syndrome (*n* = 43, 55.8%), pheochromocytoma (*n* = 26, 33.8%), and Conn’s syndrome (*n* = 8, 10.4%).

Of 116 laparoscopic operations, conversion was required in 9 cases (7.8%). The morbidity rate following 107 successful laparoscopic adrenalectomies was 3.7% (n = 4). No mortality occurred over 30 days postoperatively.

Eleven patients (9.5%) in the laparoscopic surgery group had previously undergone laparoscopic or laparotomic abdominal surgery, of whom 2 ended up with conversion.

Overall, oral intake was initiated on the first postoperative day in 126 patients, and on the second day in 4 patients due to intensive care unit stay during the first postoperative night. One patient had delayed oral intake (on day 7) due to prolonged intensive care unit stay complicated by respiratory failure. The mean duration of hospital stay was 2.4 days (range 1–14 days), the mean operative time was 79.3 min (range 40–210 min), and the mean tumor size was 4.3 cm (range 1–11 cm). The mean estimated blood loss was 49 ml (range 0–450 ml). The distribution of ASA scores was as follows: I for 32 patients (24.4%), II for 84 patients (64.1%) and III for 15 patients (11.5%).

The mean hospital stay was 1.7 days (range 1–5 days) for patients undergoing successful laparoscopic surgery, as compared with a mean of 5.3 days (range 2–14 days) for patients who underwent open surgery or who required conversion form laparoscopy.

Overall, the series included 58 left (44.3%), 45 right (34.4%), and 28 bilateral (21.3%) adrenalectomies. Table 1 summarizes details according to the type and distribution of 131 adrenalectomies. Table 2 summarizes data on successful laparoscopic adrenalectomies according to the types of lesions.

**Table 1:** Demographic features of patients and operative data according to the types and distribution of 131 adrenalectomies

	Unilateral laparoscopic			Bilateral laparoscopic	Conversion	Open
		Left	Right			
*Adrenalectomies (n)*	85	49	36	22	9	15
*Mean age, years, (range)*	50.6 (27–72)	52 (28–72)	48.8 (27–68)	39.9 (23–54)	48.9 (26–70)	45.5 (24–64)
*Female/Male* (n)	60/25	34/15	26/10	18/4	5/4	8/7
*ASA scores, I/II/III/IV (n)*	20/56/9/0	10/33/6/0	10/23/3/0	10/11/1/0	1/6/2/0	1/11/3/0
*Duration of operation, min (mean, range)*	60.4 (40–95)	60.9 (50–95)	59.7 (40–85)	130 (90–210)	110.6 (80–135)	94.6 (70–135)
*Estimated blood loss (ml)*	32.7 (0–200)	28.9 (0–150)	37.9 (0–200)	69.1 (0–450)	117 (50–250)	76 (20–200)
*Tumor size* ^*a*^ *, cm (mean, range)*	3.7 (1–8)	3.4 (1–8)	4 (1.2–7.2)	4.7 (3.2–6.2)	4.8 (3–9.3)	7.6 (4–11)
*Complications (n, %)*	2 (2.4)	–	2 (5.6)	2 (9.1)	–	1 (6.7)
*Postoperative oral intake, days (mean, range)*	1.02 (1–2)	1 (1–1)	1.06 (1–2)	1 (1–1)	1 (1–1)	1.5 (1–7)
*Postoperative hospital stay, days (mean, range)*	1.4 (1–4)	1.3 (1–4)	1.4 (1–4)	3 (1–5)	4.3 (2–10)	5.9 (4–14)

^a^In bilateral cases, the largest tumor size was considered

**Table 2:** Demographic features of patients and operative data for successful laparoscopic adrenalectomies according to the types of lesions

	Cushing’s syndrome		Pheochromocytoma		Incidentaloma		Conn’s syndrome	Other
	Unilateral	Bilateral^a^	Unilateral	Bilateral^a^	Unilateral	Bilateral^a^	Unilateral	Unilateral
*Laparoscopic adrenalectomies (n)*	26	17	25	1	17	4	8	9
*Mean age, years, (range)*	52.8 (39–68)	40.5 (26–54)	47.1 (27–64)	51	52.2 (27-68)	34.8 (23–50)	52.9 (39–72)	49.3 (33–59)
*Female/Male* (n)	22/4	15/2	14/11	1/0	13/4	2/2	5/3	6/3
*Body mass index, kg/m2, (mean, range)*	27.1 (22.8–43.0)	29.2 (23.8–39.4)	27.3 (18.8–39.1)	28.9	28.3 (20.8–38.1)	29.3 (24.2–34.3)	28.5 (21.6–36.7)	29.7 (20.6–38.7)
*ASA scores, I/II/III/IV (n)*	2/17/7/0	8/8/1/0	4/19/2/0	II	9/8/0/0	2/2/0/0	2/6/0/0	3/6/0/0
*Right / Left (n)*	10/16	–	13/12	–	9/8	–	1/7	3/6
*Duration of operation, min (mean, range)*	61.3 (50–85)	131.5 (90–210)	60.6 (40–95)	140	61.2 (40–80)	121.3 (95–140)	61.9 (55–75)	54.4 (45–70)
*Estimated blood loss (ml)*	32.3 (0–200)	73.2 (0–450)	34 (0–130)	60	26.2 (0–100)	53.8 (35–75)	27.5 (0–100)	42 (0–150)
*Tumor size* ^*a*^ *, cm (mean, range)*	3.1 (1.5–5)	4.7 (3.2–6.2)	4.2 (1.8–8)	3.5	4.3 (1.1–7.2)	4.9 (4.3–5.9)	1.6 (1–2)	4.6 (1.2–7.5)
*Complications (n, %)*	1 (3.8)	2 (11.7)	1 (4)	–	–	–	–	–
*Postoperative oral intake, days (mean, range)*	1.04 (1–2)	1 (1–1)	1.04 (1–2)	1	1 (1–1)	1 (1–1)	1 (1–1)	1 (1–1)
*Postoperative hospital stay, days (mean, range)*	1.4 (1–4)	3 (1–5)	1.3 (1–3)	3	1.5 (1–4)	2.8 (2–3)	1 (1–1)	1.4 (1–3)

^a^In bilateral cases, the largest tumor size was considered

There was a significant female preponderance among the cases (*p* < 0.05). Gender, tumor size and BMI were found to have no significant relationship with the operative time (*p* > 0.05). Cushing’s syndrome (39.7%) accounted for the largest proportion of diagnoses. In no case were simultaneous laparoscopic interventions needed for multiple diagnoses. Laparoscopic interventions included two patients with metastases (1 left, 1 right), both of which were due to lung cancers. The mean size of the metastatic lesions was 2.1 cm (1.2 and 3.0 cm). The smallest and the largest tumors removed laparoscopically were 1 cm and 8 cm in size in patients with Conn’s syndrome and pheochromocytoma, respectively.

Bilateral laparoscopic adrenalectomy was performed in 24 patients (19 females/5 males) with a mean age of 40.5 years (range 23–67 years) and a mean BMI of 29.1 kg/m^2^ (range 23.8–39.4 kg/m^2^) for Cushing’s syndrome (*n* = 19, 79.2%), incidentaloma (*n* = 4, 16.7%), and pheochromocytoma (n = 1, 4.2%). Two patients in this group ended up with conversion.

Indications for open adrenalectomy in 15 patients included malignant characteristics of the lesions (*n* = 5), previous multiple abdominal operations due to colon tumors (*n* = 2), severe chronic obstructive pulmonary disease (n = 2), difficulty in appropriate positioning of the patient due to previous surgery for disc herniation (n = 2), recurrent Cushing’s syndrome (*n* = 1), recurrent pheochromocytoma (n = 1), previous hydatid cyst operation (n = 1), and patient refusal of a laparoscopic procedure (n = 1).

Open adrenalectomies (*n* = 15) and bilateral laparoscopic adrenalectomies (*n* = 24) had statistically significant longer mean operation time than unilateral adrenalectomies (*p* values respectively 0.019 and < 0.001).

### Morbidity - conversions

Following successful laparoscopic procedures, Clavien-Dindo grade-I port-site infections occurred in four patients with Cushing’s syndrome (*n* = 3) and pheochromocytoma (*n* = 1). One patient with Cushing’s syndrome persistently had high cortisol levels due to retrocaval residual adrenal tissue, for which open surgical correction was performed two months after the initial laparoscopic procedure. There was no problem of postoperative normalization in the hormone profile of the remaining patients.

Conversion to open adrenalectomy was required in nine patients (7.8%) due to causes summarized in Table 3. The most common cause was the difficulty of dissection in three cases due to the limited space imposed by the lack of effective liver retraction. The other causes included bleeding due to injury to the left renal vein (*n* = 2), inability to locate the adrenal gland within the fatty tissue due to Cushing’s syndrome (n = 2), and severe intraabdominal adhesions secondary to previous surgery (n = 2). Risk factors (age, ASA score, BMI, tumor size, lesion type) between the cases finished laparoscopically and converted to open surgery were found not significantly different (*p* > 0,05).

**Table 3:** Causes of conversion to open adrenalectomy

Year	Diagnosis	Cause	Side	BMI^a^ (kg/m^2^)	Previous surgery
*2009*	Cushing’s syndrome	Adhesions secondary to previous surgery	Bilateral	28.6	Cholecystectomy
*2012*	Pheochromocytoma	Bleeding	Right	20.2	-
*2012*	Pheochromocytoma	Difficult dissection due to limited space	Right	29.6	-
*2012*	Incidentaloma	Bleeding	Right	29.0	-
*2013*	Incidentaloma	Difficult dissection due to limited space	Left	29.7	-
*2016*	Cushing’s syndrome	Inability to locate the adrenal gland	Bilateral	27.5	-
*2017*	Cushing’s syndrome	Inability to locate the adrenal gland	Left	26.8	-
*2017*	Incidentaloma	Adhesions secondary to previous surgery	Left	37.4	Umblical hernia
*2017*	Cushing’s syndrome	Difficult dissection due to limited space	Right	28.1	-

^a^BMI: Body mass index

**Fig. 2:**
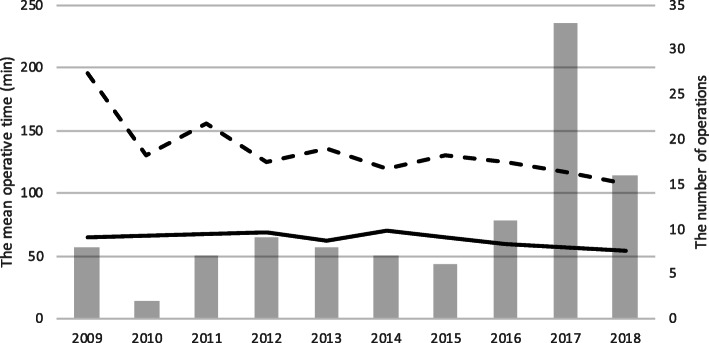
The number of operations and mean operative times according to years. The total number of operations. The mean operative time for bilateral laparoscopic adenalectomies (min). The mean operative time for unilateral laparoscopic adenalectomies (min)

The laparoscopic operative time showed notable decreases with enhanced experience and anatomical orientation (Fig. 2).

**Fig. 3:**
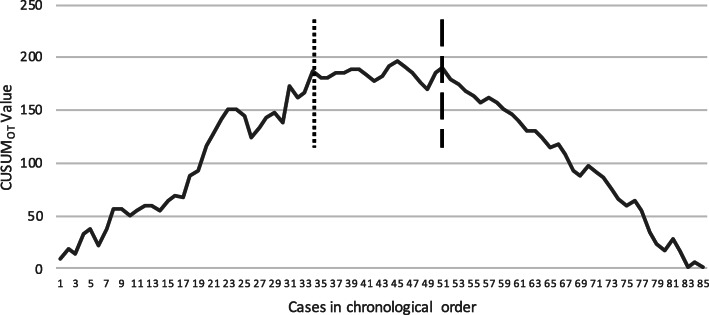
The CUSUM_OT_ chart for unilateral laparoscopic adrenalectomies. Calculated CUSUM_OT._ Operation Number 34, which ends the learning period and starts the period of acquiring competence. Operation Number 51, which ends the period of acquiring and starts the period of mastering

The learning curve for unilateral laparoscopic adrenalectomies was evaluated by the CUSUM analysis (CUSUM_OT_) in relation to the operative time. The CUSUM_OT_ graph obtained demonstrated that the elevation of the curve began to reduce and became relatively steady after the 34th operation, peaking at the 45th operation, and beginning to slope down after the 51st operation. Thus, three distinct phases could be identified, denoting the learning period (phase 1 involving operations 1 to 34), the period of acquiring competence (phase 2 involving operations 35 to 51), and finally the period of mastering (phase 3 involving operations 52 to 85) (Fig. 3).

The learning curve for bilateral laparoscopic adrenalectomies could not be assessed because of the inadequate number of cases.

## Discussion

The current study presents an overall analysis of the experience gained from laparoscopic adrenalectomy operations over a nine-year period from the standpoint of a highly specialized surgeon in endocrine surgery. Laparoscopy has proved to be as effective as laparotomy and is currently considered to be the gold standard for the surgical treatment of adrenal pathologies [4, 9, 13, 14].

Several minimally invasive laparoscopic approaches have been described for adrenalectomies, including transperitoneal, lateral retroperitoneal, and posterior retroperitoneal approaches [15]. In our center, the first laparoscopic procedure was performed in 1995 with the use of the lateral transabdominal approach, which in turn formed the basis of education and training. Being a high-volume center for laparoscopic operations played a contributory role in improving the technique, as well. Vrielink et al. emphasized the importance of the volume of procedures performed in a surgical unit and pointed out the difficulty in achieving competency in the setting of an annual case-volume insufficient to complete the learning curve [15]. Approximately 20–40 procedures are required to reach the learning curve in order to gain sufficient experience and proficiency [16–18]. Although the posterior retroperitoneal approach was favored by Walz et al. due to some advantages such as provision of a direct route to the adrenal glands with fewer dissections, avoidance of peritoneal adhesions secondary to previous operations, and obviation of repositioning of patients with indications for a bilateral adrenalectomy [19], several limitations have rendered this approach less popular, including the unfamiliar anatomical environment, a smaller working space, and a longer learning curve [15].

In our study, the candidate surgeon specialized in endocrine surgery was trained with complete adherence to a rigorous program implemented by a competent mentor. Based on the literature data, completion of the first 30 cases was assumed to satisfy the fulfilment of the learning curve [16, 17]. The CUSUM_OT_ graph derived in relation to the operative time clearly showed that the steepness of the slope did not transform into a relatively smooth plateau until after the 34th operation. Our findings confirm that laparoscopic transabdominal adrenalectomy can be safely completed after completion of the learning curve. In a multicenter study evaluating the surgical learning curve for posterior retroperitoneoscopic adrenalectomy, Vrielink et al. reported a range of 24–42 procedures for the achievement of competency [15]. In our single-center study, the range of the learning curve for lateral transperitoneal adrenalectomy operations was somewhat similar to their findings.

In the light of our experience, a left adrenalectomy seems to be a more difficult operation than a right adrenalectomy due to the absence of major anatomical landmarks such as the inferior vena cava and the presence of smal-sized main vessels, retroperitoneal fat and the pancreatic tail encroaching on the surgical space. Therefore, thanks to the experience gained and enhanced familiarization with anatomical orientation from high-volume laparoscopic cholecystectomy procedures, surgical training involved right adrenalectomies to begin with. In contrast, Sommerey et al. preferred left adrenalectomy for the beginning of surgical training because they considered right adrenalectomy to be more challenging due to an increased bleeding risk through the inferior vena cava or the possibility of an inadvertent injury to the adrenal capsule [18].

We currently remove tumors larger than 4 cm and those that are smaller, but hormonally active or suspected of being malignant on computed tomography. In our laparoscopic series, Cushing’s syndrome accounted for more than half of the resections (55.8%) for functional tumors. The rates of conversion (7.8%) and morbidity (3.7%) were low and similar to those reported in the literature [11, 14, 18]. The main inconsistency with the reported series was in the operative time, with a mean of 60.4 min in unilateral laparoscopic procedures [3, 18]. Among 9 cases of conversion, 44.4% had Cushing’s syndrome, followed by incidentaloma with 33.3%. The reasons for conversion were inability to locate the adrenal gland, difficulty in dissection due to limited space, adhesions secondary to previous surgery, and inadvertent damage to the adrenal gland caused by the rigidity of laparoscopic devices. Pheochromocytoma is more often implicated among the reasons for conversion [3, 7], probably due to technical problems associated with hypervascularization. In our series, conversion was required in 2 (7.1%) of 28 patients with pheochromocytoma due to uncontrolled bleeding that occurred during the early ligation of the adrenal vein in one case, and the development of adrenal crisis when limited space rendered dissection difficult in the other. Therefore, successful and safe resection of pheochromocytoma lesions requires a combination of individual experience, cooperation with the anesthesiology team, and appropriate medical treatment and preparation in the preoperative period.

The presence of previous laparotomic surgeries may not be considered a contraindication to laparoscopic adrenalectomy [3]. Among 11 patients with a history of previous surgeries for various reasons, conversion was required in only 2 patients (18.1%) mainly due to adhesions.

Contrary to our expectations, we did not find any relationship between tumor size and operative time in successfully completed laparoscopic adrenalectomies, where all the tumors were 8 cm or smaller in diameter. Castillo et al. reported significantly longer operative time, increased blood loss, and longer hospital stay in large adrenal masses (≥8 cm), without an adverse effect on perioperative morbidity [20]. In contrast, Shen et al. found in their multivariate analysis that a tumor size of ≥5 cm was the most important independent predictive factor for conversion [21].

The only relative contraindication to laparoscopic adrenalectomy is primary adrenocortical carcinoma, where there is a substantial risk for positive margins and intraoperative tumor spillage [22]. In this respect, we also advocate laparotomy for complete resection and adequate lymphadenectomy. As for other malignancies, successful laparoscopic resection is feasible as seen in two of our cases with lung metastases. Laparoscopic removal of adrenal metastases performed by expert surgeons has been shown to be as safe as open procedures, with noninferior oncologic results [23, 24].

A higher BMI has been reported to complicate laparoscopic adrenalectomies [25], though we did not find any relationship between BMI and operative time in our cases whose BMI ranged between 18.8 and 43 kg/m^2^. Similarly, Inaishi et al. found no significant differences in peri- and postoperative outcomes of transabdominal laparoscopic adrenalectomy between patients with and without obesity [26].

## Conclusion

Laparoscopic adrenalectomy procedures require a dedicated team involving experienced endocrine surgeons who have achieved competency after completion of the learning curve. To improve the outcomes, patients with adrenal pathologies should be referred to high-volume surgical centers with specialized endocrine surgeons experienced in laparoscopic adrenalectomies.

## Data Availability

The datasets used and/or analysed during the current study available from the corresponding author on reasonable request.

## Abbreviations

BMI: Body Mass Index
CUSUM: Cumulative Sum
CUSUM_OT_: Cumulative Sum (Operation Time)
LA: Laparoscopic Adrenalectomy
LTA: Laparoscopic Transperitonal Adrenalectomy
ST: Initials of Serkan Teksoz, M.D. FEBES
